# Chimeric antigen receptor engineered natural killer cells for cancer therapy

**DOI:** 10.1186/s40164-023-00431-0

**Published:** 2023-08-10

**Authors:** Yalan Zhang, Weilin Zhou, Jiangping Yang, Jinrong Yang, Wei Wang

**Affiliations:** 1 Department of Biotherapy, State Key Laboratory of Biotherapy and Cancer Center, West China Hospital, Collaborative Innovation Center for Biotherapy, Sichuan University, Chengdu, 610041 People’s Republic of China; 2 Department of Head and Neck Oncology and Department of Radiation Oncology, Cancer Center and State Key Laboratory of Biotherapy, West China Hospital, Sichuan University, Chengdu, 610041 Sichuan People’s Republic of China; 3 Hematology Research Laboratory, Department of Hematology, State Key Laboratory of Biotherapy and Cancer Center, West China Hospital, Sichuan University, Chengdu, 610041 Sichuan People’s Republic of China

**Keywords:** NK cells, CAR-NK, Immunotherapy, Cancer treatment, Preclinical studies, Clinical trials

## Abstract

Natural killer (NK) cells, a unique component of the innate immune system, are inherent killers of stressed and transformed cells. Based on their potent capacity to kill cancer cells and good tolerance of healthy cells, NK cells have been successfully employed in adoptive cell therapy to treat cancer patients. In recent years, the clinical success of chimeric antigen receptor (CAR)-T cells has proven the vast potential of gene-manipulated immune cells as the main force to fight cancer. Following the lessons learned from mature gene-transfer technologies and advanced strategies in CAR-T therapy, NK cells have been rapidly explored as a promising candidate for CAR-based therapy. An exponentially growing number of studies have employed multiple sources of CAR-NK cells to target a wide range of cancer-related antigens, showing remarkable outcomes and encouraging safety profiles. Clinical trials of CAR-NK cells have also shown their impressive therapeutic efficacy in the treatment of hematological tumors, but CAR-NK cell therapy for solid tumors is still in the initial stages. In this review, we present the favorable profile of NK cells as a potential platform for CAR-based engineering and then summarize the outcomes and strategies of CAR-NK therapies in up-to-date preclinical and clinical investigations. Finally, we evaluate the challenges remaining in CAR-NK therapy and describe existing strategies that can assist us in devising future prospective solutions.

## Introduction

Immune cells serve as the pillar of strength in antitumor and antiviral processes [1]. Through their rapid recognition and lysing of nascent transformed cells, immune cells can prevent tumorigenesis in the initial stage [2]. However, once malignant cells proliferate and metastasize uncontrollably, they change and depress the immunological responses mediated by host immune cells [3]. By infusing functionally active effector cells into immunocompromised patients, a process known as adoptive cell therapy (ACT), we can reconstruct host immunity and provide a promising strategy for disease treatment [4, 5]. Adoptive transfer of autologous immune cells that have been activated and amplified ex vivo has shown encouraging efficacy in patients with certain hematological cancers. However, the therapeutic efficiency in other tumors is far from satisfactory [6, 7]. With the advancement of gene engineering technology, cytotoxic T cells have been equipped with CARs, which endow T cells with superior and more precise killing capacity. In recent years, CAR-T cells have achieved numerous breakthroughs in cancer treatment, especially in hematologic malignancy treatment [8–14]. A multitude of CAR-T investigations regarding cancer treatment have progressed into the clinical trial stage, with a high rate of complete remission (CR) being exhibited, and some CAR-T cells have even developed into commercial products [15]. To date, six CAR-T products for treating hematological tumors have been approved by the US Food and Drug Administration (FDA), including Kymriah (Novartis), Yescarta (Gilead), Tecartus (Gilead), Breyanzi (Bristol Myers Squibb), Abecma (Bristol Myers Squibb and Bluebird Bio), and Carvykti (Legend and Janssen). CD19 (four products) and B-cell maturation antigen (BCMA) (two products) are the two primary antigens targeted by CAR-T cells to treat relapsed/refractory (R/R) B-cell-derived leukemia, lymphoma, and multiple myeloma [9, 13, 16–21]. Despite these promising outcomes of CAR-T cells in the treatment of hematological tumors, their limited efficacy in the treatment of solid tumors necessitates the exploration of novel strategies to help CAR-T cells break the barriers in solid neoplasm. CAR-T immunotherapy requires apheresis and time-consuming expansion of autologous immune cells from patients. For some patients with aggressively progressing cancer, costly and complicated procedures may result in delayed therapy. In addition, heavily pretreated cancer patients are unable to provide sufficient normal T cells, creating an additional barrier to CAR-T-cell development. Therefore, a surge of interest has recently focused on seeking other candidate immune cells to be engineered with CARs [22].

NK cells, a subset of innate lymphoid cells (ILCs) with diversified killing mechanisms, have recently become a focal point in the application of immunotherapy. The function of NK cells is regulated by a sophisticated array of activating and inhibitory receptors that can distinguish between healthy cells and transformed cells. The integrated signals from the engagement of these receptors and ligands can determine whether NK cells initiate killing activities against aberrant cells or maintain their tolerance of healthy cells [23, 24]. In contrast to T cells, NK cells recognize cancer cells in a human leukocyte antigen (HLA)-unrestricted manner, resulting in the lowest possibility of graft versus host disease (GVHD) development [25]. Furthermore, NK cells rarely induce severe toxicities such as cytokine release syndrome (CRS) and immune effector cell-associated neurotoxicity syndrome (ICANS) in vivo [26]. Owing to these favorable attributes, NK cells engineered with CARs can overcome many hurdles that prevent CAR-T therapy from further application. The successful adoptive transfer of allogeneic NK cells into patients further identifies NK cells as a promising platform for CAR engineering and as “off-the-shelf” products for wide application [22]. The strategies of CAR design and transduction used in CAR-T therapy are applicable in NK engineering with encouraging outcomes. To date, CAR-NK cells have shown impressive efficacy in the treatment of hematological tumors and have been widely studied in the treatment of solid tumors, with numerous breakthroughs, such as in the treatment of glioblastoma, breast cancer, and ovarian cancer [27–29]. In this review, we present the favorable profile of NK cells as a potential platform for CAR-based engineering and then summarize the outcomes and strategies of CAR-NK cell therapy in up-to-date preclinical and clinical investigations. Finally, we evaluate the challenges remaining in CAR-NK cell therapy and describe existing strategies that can assist us in devising future prospective solutions.

## An overview of NK cell biological properties

### Development and classification

NK cells are a subgroup of innate lymphoid cells (ILCs) and are identified as the first line of defense against virally infected and/or transformed cells [30]. Derived from CD34^+^ hematopoietic progenitor cells in bone marrow, NK cells develop in a continuous process in bone marrow as well as in some secondary lymphoid organs (SLOs), such as the spleen, tonsils, thymus, and liver [31, 32]. However, it is unclear whether NK cells differentiate in a linear or nonlinear manner [33]. The developmental stages of NK cells differ significantly among different anatomical locations. Immature NK cells are predominantly distributed in lymph nodes and intestines and have tissue-adaptation signatures, whereas terminally differentiated NK cells mainly populate the blood, bone marrow, spleen, and lungs and have improved effector function [34]. According to the expression levels of CD56 and CD16, NK cells are divided into two major subgroups: CD56^bright^CD16^−^ and CD56^dim^CD16^+^ NK cells [35]. CD56^bright^ NK cells are immature populations and are mainly distributed in SLOs. They were previously thought to be involved in immunomodulation, but recently, they have been identified with robust cytokine-releasing potential after priming with proinflammatory cytokines such as interleukin-15 (IL-15). CD56^bright^ NK cells are more similar to helper cells, secreting abundant cytokines such as interferon-γ (IFN-γ), tumor necrosis factor-β (TNF-β) and granulocyte–macrophage colony-stimulating factor (GM-CSF) [36, 37]. CD56^dim^ NK cells represent the final stage of NK cell maturation and constitute approximately 90% of circulating NK cells. The increased expression of CD16a (FcγRIIIa) and cytotoxic molecules in CD56^dim^ NK cells allows them to mediate serial killing activities toward malignant cells, for example, via antibody-dependent cellular cytotoxicity (ADCC) and death receptor-mediated apoptosis [38, 39]. Recently, high-resolution sequencing technologies further revealed increased heterogeneity of NK cells in different organs, indicating that more NK subpopulations can be further defined beyond the simple delineation of CD56^bright^CD16^−^ and CD56^dim^CD16^+^ NK cells [34, 40, 41].

### Activation and cytotoxicity

**Fig. 1:**
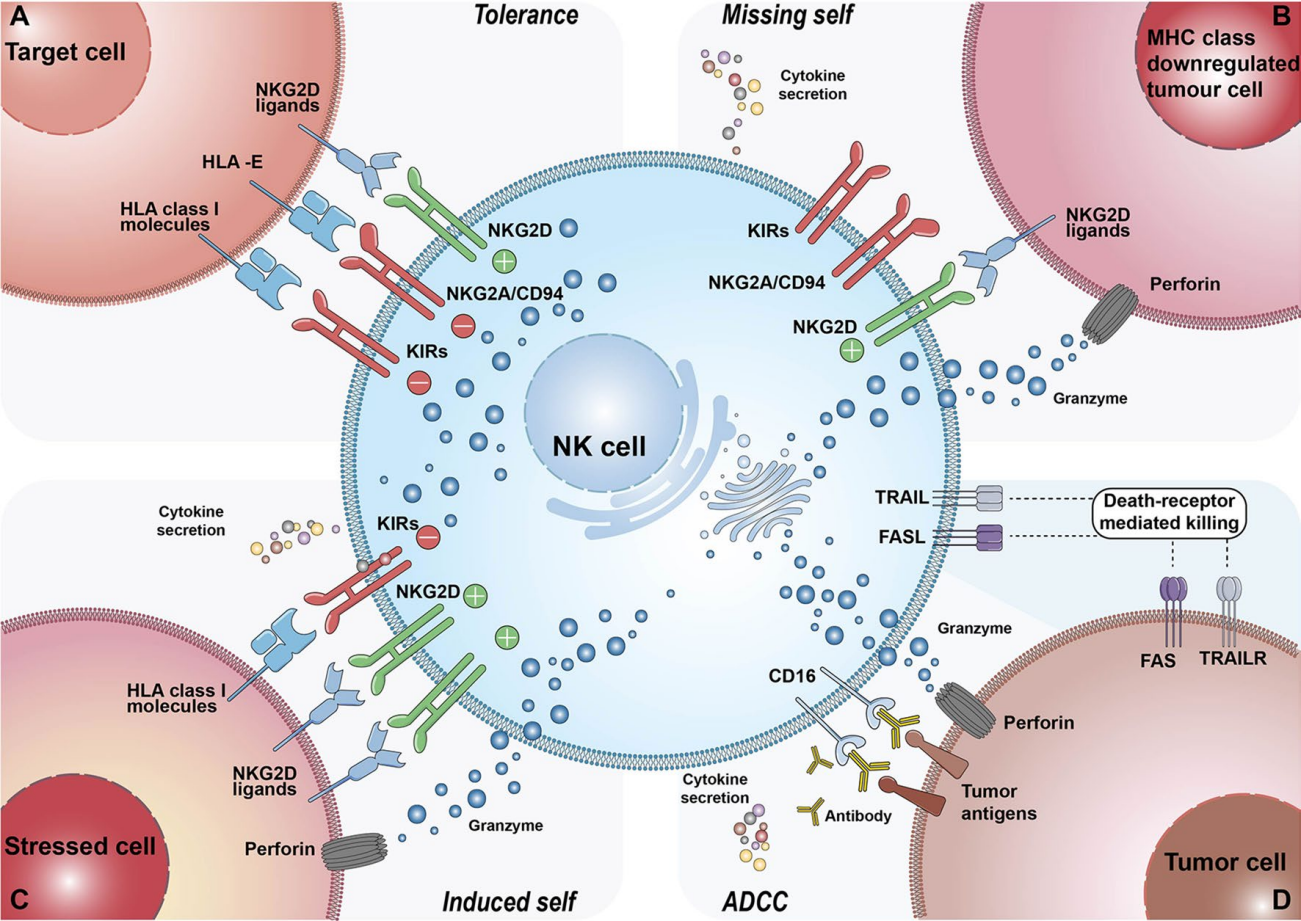
The mechanism of NK activation and self-tolerance. **A** In healthy conditions, self-HLA class I molecules of healthy cells bind the inhibitory receptors of NK cells such as KIRs and NKG2A/CD94. Dominant inhibitory signaling suppressed the cytolytic ability of NK cells to make autologous healthy cells “licensed”. **B** The majority of tumor cells downregulate or lost their MHC-I molecule expression to escape from the immune cells attacking. This results in decreasing tumor ligands combining with inhibitory receptors of NK cells, thus NK cells are activated to secret perforin and granzyme to lyse tumor cells. **C** Overexpressed activating ligands on stressed cells engage with NK cell receptors, leading to superior activating signaling surpassing inhibitory signaling. As a result, NK cells transform into activation state and initiate cell lysing. **D** Antibody-dependent cell-mediated cytotoxicity, ADCC. The tumor-specific Fc fragment binds CD16 (FcγRIII) of NK cells, resulting in ADCC development. In addition to ADCC, other killing mechanisms of NK cells include death-receptor-mediated and perforin/granzyme-mediated killing activities

NK cells are critical for immune surveillance and antitumor responses in vivo. These biological functions are regulated by integrated signals from the stochastically expressed activating and inhibitory receptors on NK cells [42]. The activation and inhibition mechanisms of NK cells are depicted in Fig. 1. Ubiquitously expressed major histocompatibility complex class I (MHC-I) molecules (also known as HLA class I) on healthy cells can bind the inhibitory killer cell immunoglobulin-like receptors (KIRs) or NKG2A of NK cells, which can deliver predominant inhibitory signaling to maintain NK “self-tolerance” [24, 43]. However, tumor cells often downregulate their MHC-I molecule expression to evade the attack of CD8^+^ cytotoxic T cells, as the target recognition of CD8^+^ T cells relies on antigen presentation by MHC-I [44, 45]. Thus, the signaling balance of NK cells is broken, and they are inclined toward an activation state (also known as the “missing-self mechanism”). Other transformed or stressed cells expressing excessive activating ligands, such as NKG2D ligands, can directly stimulate NK cell activation through receptor‒ligand engagements called immune synapses [46]. The formulation of immune synapses can initiate firm adhesion and enable the focused delivery of lytic granules such as perforin and granzymes onto the target cells, inducing the apoptosis of target cells [45, 47]. Significantly, a single degranulation can be sufficient to lyse a target cell [48]. Following early lytic granule-mediated killing activities, delayed cell apoptosis responses can be initiated by target cells engaging with death ligands expressed on NK cells such as Fas ligand (Fasl) and TNF-related apoptosis-inducing ligand (TRAIL), conferring the serial killing ability of NK cells [49]. Additionally, CD16 is a potent activating receptor that allows NK cells to engage with antibody-opsonized target cells through ADCC. This crosslinking interaction can subsequently induce NK cells to release the cytotoxic substances mentioned above [50]. In addition to triggering their powerful killing ability, NK cells can secrete an array of cytokines and chemokines to stimulate broader cellular immune responses. For example, the IFN-γ and TNF released by activated NK cells can synergistically mediate the death of target cells [51]. IFN-γ can not only directly activate macrophages but also indirectly promote CD8^+^ T-cell-mediated immune responses by elevating MHC-II molecule expression on antigen-presenting cells [52]. The NK cell-dendritic cell axis also plays a critical role in tumor immunity. CCL5, XCL1, XCL2, and FLT3L secreted by NK cells are the major chemokines that recruit conventional type 1 dendritic cells (cDC1s). cDC1s can present tumor-associated antigens (TAAs) from apoptotic tumor cells to CD4^+^ and CD8^+^ T cells, thus inducing potent T-cell-mediated immune responses [52–54].

## The strength of NK cells as immunotherapy candidates

### Accessibility to abundant cell sources

**Fig. 2:**
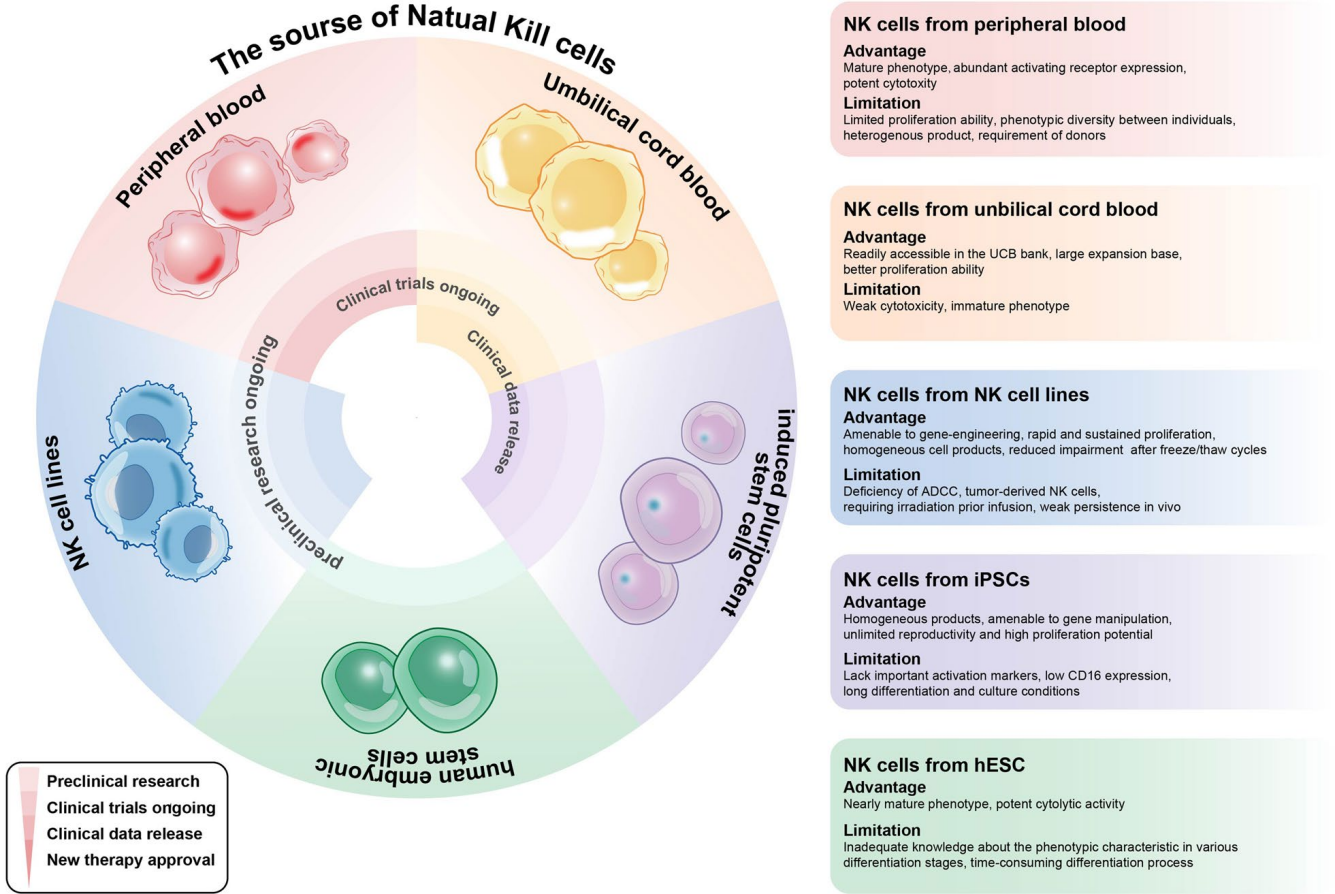
The research progress, advantages, and limitations of various NK cell sources. NK cells can be obtained from 5 different sources: PB, UCB, iPSC, hESC, and NK cell lines. Most cell sources have remarkable tumor-eliminating ability and provide clinically meaningful benefit, having transitioned into in-human studies of different stages. Each source of NK cells has its own set of strengths and limitations

NK cells can be obtained from autologous and allogenic sources. Initially, autologous NK cells were the major alternative in adoptive cellular therapy owing to their safety [55, 56]. The evidence suggests that autologous NK cells are not sufficient to exert robust antitumor responses, in part due to the NK inhibitory effects mediated by self MHC-I molecules and functional impairments caused by prior heavy treatment [57, 58]. These findings encourage transitioning the focus on autologous NK cells to allogenic NK cell sources, the use of which can avoid cumbersome collection processes and satisfy clinical doses [22, 59]. These NK cell sources include peripheral blood (PB), umbilical cord blood (UCB), NK cell lines, and stem cell-derived NK cells [42]. Each source of NK cells has its own set of strengths and limitations, as summarized in Fig. 2.

PB-derived NK cells, obtained through donor lymphocyte apheresis, represent a conventional option in CAR-NK investigations of cancer treatment. PB-NK cells are mainly a mature population characterized by CD56^dim^CD16^bright^ cells, without obvious individual variations [60]. Additionally, PB-NK cells show relatively abundant expression of activating receptors such as NKG2D, NKp44, and NKp46, which significantly foster NK cell destruction potential against malignant cells [29]. However, the low proportion of NK cells in PB (approximately 10–15%) largely hinders the cell collection and ex vivo expansion process [34, 61]. NK cells isolated from PB are in various maturation stages and thus are characterized by heterogeneous receptor expression profiles, from maturing to fully mature phenotype variation [62, 63]. Thus, the standardization and stability of cell products are hard to guarantee.

UCB-NK cells are also a valuable and well-studied source, constituting up to 30% of UCB lymphocytes [64]. UCB-NK cells are easy to collect and can be frozen in a cell bank; thus, the incumbrances associated with the apheresis of healthy donors and time-consuming amplification can be avoided [65]. There are fewer contaminating T cells among UCB-NK cells. Furthermore, cell sorting techniques such as immunomagnetic cell separation can assist in attaining high-purity NK cells, minimizing the risk of GVHD as much as possible [66–68]. Additionally, UCB cells offer abundant cell sources, where hematopoietic stem cells and progenitor cells can be acquired and then differentiate into therapeutic NK cells with favorable phenotypes [69, 70]. However, compared to PB-derived NK cells, UCB-NK cells possess relatively weak cytotoxic abilities against malignant cells owing to their natural immature phenotype, represented by the CD56^−^CD16^+^ population [71, 72]. In addition, UCB-NK cells express a lower level of activating receptors and adhesion molecules (such as CD16, CD2, and CD11a) [73] and a high level of the inhibitory receptor NKG2A [74]. This feature calls for sufficient ex vivo stimulation to promote a more mature state of UCB-NK cells, thus augmenting their cytotoxicity and persistence [75].

In view of the delay in collection and complex expansion of primary cells, focus has increasingly transitioned to immortalized NK cell lines such as NK-92 [76, 77], NK-92MI [78, 79], KHYG-1 [80], and YTS [81]. NK-92 has been the most extensively studied cell line in NK-based clinical trials [82]. As they mostly lack KIR expression, NK-92 cells are more sensitive and robust in their response to tumor cells [83]. NK-92 cells are homogenous and easy to mount in desirable quantities. In addition, they can be easily genetically engineered under good manufacturing practice (GMP)-compliant methodologies, representing the industry-transformation potential of NK-92 cells. However, NK-92 cells are aneuploid and of malignant origin, thus requiring irradiation before cell infusion. Irradiation can limit the persistence of NK-92 cells and negatively impact their durable therapeutic efficacy [84]. NK-92 cells are naturally deprived of CD16, indicating a deficiency in the ADCC response [85]. Recently, high-affinity CD16 variant molecules were successfully engineered on NK-92 cells to perform a more comprehensive and robust effector function [86].

Recently, there has been increased interest in stem cells such as human embryonic stem cells (hESCs) and induced pluripotent stem cells (iPSCs) as sources of NK cells, which hold great potential to be standardized “off-the-shelf” therapies. Through multiple rounds of stromal cell coculturing or cytokine cocktail stimulation, iPSCs or hESCs can gradually develop into mature and homogeneous CD45^+^CD56^+^ NK cells [87–89]. Phenotype analysis has demonstrated that hESC-derived NK cells can differentiate toward nearly mature phenotypes akin to PB-NK cells [89, 90]. A study showed that hESC-NK cells have more cytolytic effects on tumor cells than UCB-NK cells [91]. iPSC-derived NK cells are weak in ADCC due to a low level of CD16 expression, although they are amenable to genetic manipulation with a high-affinity CD16 molecule to restore their ADCC mechanism [92]. Relying on their excellent properties, iPSC-derived NK cells have been investigated in a large number of preclinical and clinical studies, either as a single therapy or in combination with agents, which remedies the limitations caused by their defective phenotype and killing ability [92–95]. The unlimited reproductivity and plasticity of iPSC-NK cells offer exceptional advantages for them to be potent standardized products in a broad range of applications [96, 97]. Fate therapeutics have led the way to engineer multifunctional iPSC-NK cells, with five products being evaluated in clinical settings to treat hematological and solid malignancies [68].

### Robust antitumor responses and preferable safety profile

NK cells have great potential to be broadly applied in cancer treatment. Hematological tumor cells are more accessible to NK cells and are sensitive to their responses. In the context of solid tumor cells, circulating NK cells must extravasate from the blood and traverse the tumor stroma to reach tumor beds, guided by the chemokines secreted by NK cells and other immune cells [98]. Upon entry into the tumor sites, the integrated activation signaling from NK-tumor interactions induces a series of killing activities, from cytolytic granule release and death receptor‒ligand interactions to ADCC [99]. During these processes, continuous secretion of cytokines such as IFN-γ, TNF, GM-CSF, M-CSF, IL-5, and IL-10 assists in tumor elimination by recruiting and regulating the antitumor responses of other immune cells [100]. In addition, NK cells have been identified with memory-like functions in multiple studies, which is not an attribute of innate immune cells [101, 102]. Memory-like NK cells can initiate a more rapid and robust response characterized by enhanced IFN-γ secretion. A generation scheme of memory-like NK cells that are preactivated by IL-12, IL-15, and IL-18 has been widely adopted. This special NK population has been utilized to augment and consolidate hematopoietic cell transplantation (HCT) in clinical settings, achieving promising outcomes [103–106]. A commercial memory-like NK product (WU-NK-101) developed by Wugen has also been evaluated in clinical trials (NCT05470140), showing that innovation in NK-based therapy is ongoing [107].

In addition to multiple powerful killing activities, the excellent safety performance of NK cells is another major asset making them potential immunotherapy candidates. NK cells can recognize pathologic cells in a non-HLA-restricted modality without the risk of GVHD development [108, 109] and can spare healthy cells from attacks through a “missing-self” mechanism mediated by the predominant signaling of iKIRs and NKG2A, as described above [110, 111]. The proinflammatory cytokines IL-1, IL-6, and TNF-, which are associated with cytokine storm and neurotoxicity, are also secreted at low levels by NK cells [29, 112]. A clinical study reported that ex vivo activated NK cells lasted 7 to 22 days upon infusion into patients. The short persistence of NK cells may raise doubts about durable therapeutic efficacy, but some researchers consider it an indication of controllable therapy, and the treatment efficacy may be addressed by multiple infusion doses [102]. These safety attributes of NK cells open the way for their broad application in allogeneic settings, showing the potential of these cells as off-the-shelf cellular therapy products.

### Chimeric antigen receptor (CAR) design for NK cells

**Fig. 3:**
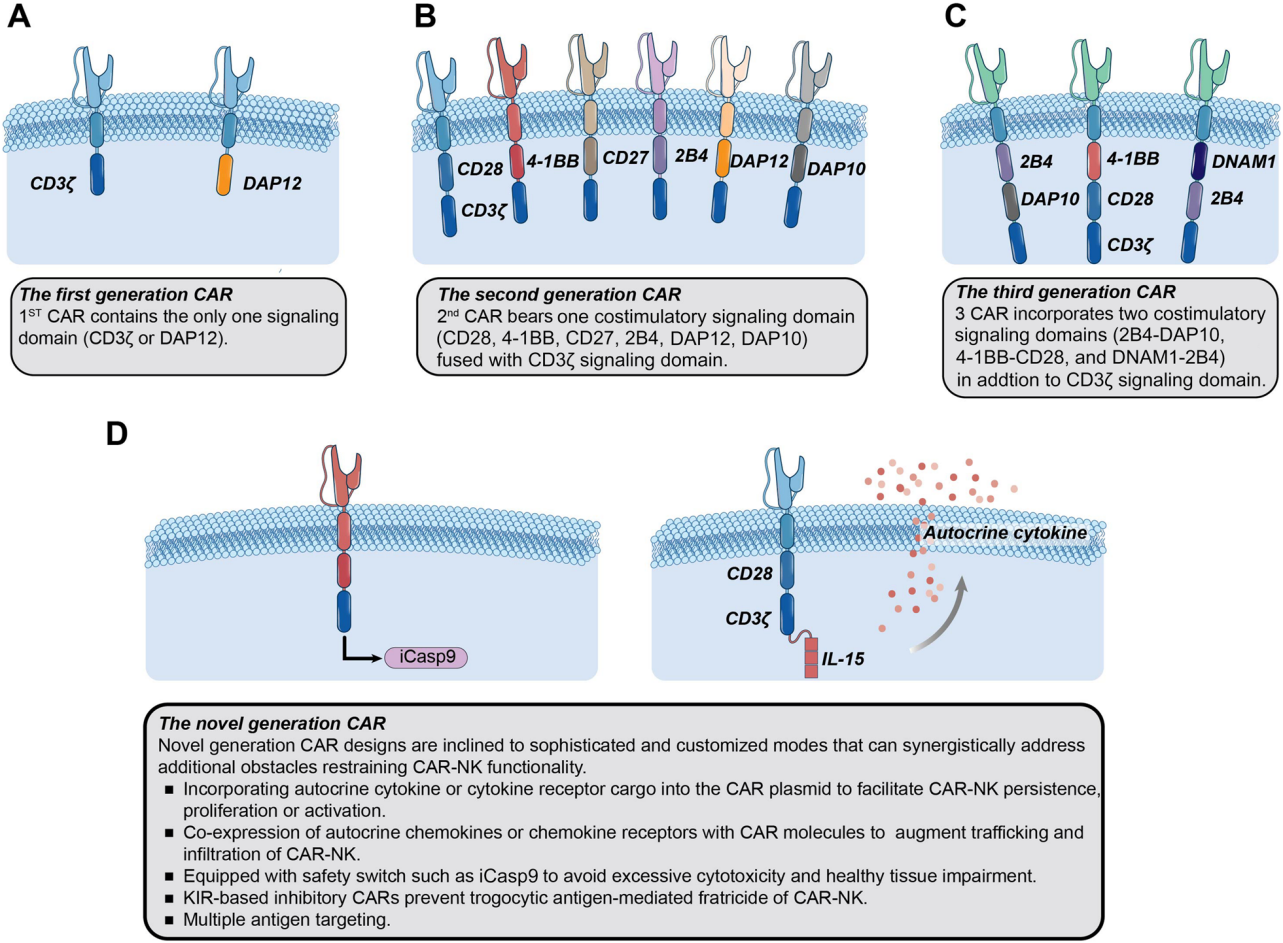
The evolution of CAR design and emerging strategies on CAR-NK structure. The main distinction of the three generation CARs lies in the number and composition of intracellular domains. In addition to the T-cell based signaling domains, NK-specific receptor (such as DAP10, DAP12, 2B4) has introduced into NK cells to explore CAR-NK therapy. The novel generation CAR strategies utilize the fundamental principles of CAR signaling and innovative approaches to enhance cytotoxicity persistence, trafficking, and safety performance of CAR-NK cells, endowing them multifunctional attributes

CAR was first introduced in T cells to endow them with target-specific recognition ability and potent killing responses [113]. CAR is a synthetic protein with three major parts: the extracellular domain, transmembrane region, and intracellular domain. The extracellular domain is composed of an antibody-derived single chain variable fragment (scFv) for antigen recognition [114, 115]. Recently, a single variable domain on a heavy chain (VHH) characterized by small size and high affinity has also been utilized for this aim [116, 117]. The hinge region connects scFv or VHH with the transmembrane region, which docks the ectodomain region of the CAR molecule to the cell membrane [118]. Intracellular signaling domains are derived from the signal transduction domains of TCRs or other activating receptors and are responsible for stimulating downstream pathways and activating CAR-carrying effector cells upon the recognition of target antigens [119, 120]. According to the number and components of the intracellular portion, CARs are traditionally classified into three generations [121] (as depicted in Fig. 3). First-generation CAR possesses only one signaling domain (usually CD3ζ) that is considered insufficient to induce a potent killing response in the absence of costimulatory domains [122, 123]. Thus, second-generation and third-generation CARs are respectively engineered with one or two costimulatory domains, such as CD28, CD137 (4-1BB), or CD134 (OX40), fused to CD3ζ, contributing to enhanced activation of effector cells [124]. However, there is no definite conclusion indicating that third-generation CAR outperforms the-second-generation CARs. Defects in CAR-effector cells, such as weak persistence and potential toxicity, promoted the development of novel-generation CARs (also known as fourth-generation CAR). Leveraging the advances in synthetic biology, innovative modules have been exploited to program CAR-engineered systems with self-supporting and safety modulation. Cytokine genes have been incorporated into CAR cassettes to support the activation and persistence of CAR-effector cells, either in autocrine or membrane-bound forms [125–127]. The inducible caspase 9 (iCasp9) suicide gene system serves as a “safety switch” that can induce the apoptosis of effector cells after the addition of small molecule drugs. iCasp9-incorporating CAR has been demonstrated efficient in controlling the toxicity of effector cells under unfavorable circumstances [26, 128] (as depicted in Fig. 3). Based on the lessons learned from current preclinical and clinical investigations, more sophisticated strategies have been developed to overcome the obstacles that hinder the efficacy of CAR-based therapy. For example, antigen escape is a major barrier to CAR-therapy and correlates with a poor prognosis [129]. A bispecific CAR that contains either two separate CARs targeting different antigens or a single CAR with two target-recognition domains can be a feasible modality to enhance the stringency of tumor recognition and prevent tumor evasion [130]. In addition, trogocytosis is an active process characterized by the transfer of surface molecules from target cells to effector cells. Trogocytosis can lead to the fratricide and dysfunction of CAR-T cells, with the potential for antigen-low tumor relapse [131]. Therefore, an inhibitory CAR (iCAR) directed to NK-cell-specific inhibitory receptors was introduced in NK cells to initiate a “don’t kill me” signal. The cooperation of tumor-targeting activating CAR (aCAR) and NK self-recognizing iCAR has demonstrated effective prevention of trogocytosis-mediated NK fratricide and enhanced CAR-NK cell activity [132].

Recently, more insights have been gained into NK cells as an alternative for CAR-targeted immunotherapy [133]. As some activating signaling moieties are shared between T cells and NK cells, such as CD3ζ, CD28, and 4-1BB, CARs conventionally designed for T cells are theoretically applicable for NK cells and have been proven effective [26, 134]. Following these encouraging outcomes, the substitution of intracellular elements of CAR with NK-biology-pertinent signaling domains has increasingly garnered interest. Some studies have adopted NK-associated DNAX-activation protein 10 (DAP10) or DAP12 as a signaling domain in place of CD3ζ [135–138]. In a comparative analysis, DAP12-based CAR resulted in an in vitro cytotoxicity of PB-derived NK cells that was superior to that of CD3ζ-based CAR [139]. Li et al. [96] assessed the functionality of nine CAR constructs (one T-like CAR and eight NK-like CARs) based on the NK-92 cell line and iPSC-derived NK cells. Several cytotoxicity assessments revealed that using the CAR containing the NKG2D transmembrane domain along with the NK-specific 2B4 costimulatory domain can endow the NK cells with the most potent killing ability and activation degree. Overall, these findings indicate that the antitumor capabilities can be further augmented based on the optimization of the CAR-NK design.

## The application of CAR-NK therapy in cancer treatment

### CAR-NK therapy for hematological tumors

The paradigm-shifting success of CAR-T therapy has provided valuable guidance for CAR-NK therapy. Initial investigations of CAR-NK cells primarily focused on the treatment of a variety of hematological cancers, especially B-cell derived malignancies (Table 1), which is identical to the preliminary stage of CAR-T cells [140]. CAR-NK cell therapy research continues to experience tremendous growth, with many strategies advancing from preclinical studies into the clinical stage. The clinical trials of CAR-NK therapy are summarized in Table 3.

**Table 1:** Overview of preclinical studies based on CAR-NK cell therapy for hematological malignancies

Disease	Target	NK cell source	Intracellular domain	Transduction methods	References
B-cell malignancies	CD19	PB-NK	CD28.CD3ζ	Retrovirus	[141]
B-cell malignancies	CD19	PB-NK	4-1BB.CD3ζ	Retrovirus	[142]
B-cell malignancies	CD19	CB-NK	CD28.CD3ζ+IL-15	Retrovirus	[128, 143]
B-cell malignancies	CD19; CD20	NK-92	CD3ζ	Retrovirus	[144]
B-cell malignancies; acute myeloid leukemia	CD19; CD276	NK-92	CD28.CD3ζ	Retrovirus	[145]
B-cell lymphoma; multiple myeloma	CD19; BCMA	NK-92	4-1BB.CD3ζ	Electroporation	[76]
B-cell acute lymphoblastic leukemia	FLT3	NK-92	CD28.CD3ζ	Lentivirus	[146]
B-cell malignancies	CD20	NK-92	CD3ζ	Retrovirus	[147]
B-cell non-Hodgkin lymphoma	CD20	PB-NK	4-1BB.CD3ζ	Electroporation	[148, 149]
T-cell malignancies	CD3	NK-92	4-1BB.CD28.CD3ζ	Lentivirus	[150]
B-cell malignancies T-cell malignancies	CD4	NK-92	CD28.4-1BB.CD3ζ	Lentivirus	[151]
T-cell malignancies	CD3; CD5	NK-92	2B4.CD3ζ/CD28.CD3ζ	Retrovirus	[152]
T-cell malignancies	CD5	NK-92	4-1BB.CD28.CD3ζ	Lentivirus	[153]
T-cell malignancies	CD5	NK-92	CD28.CD3ζ	Lentivirus	[154]
T-cell malignancies	CD5	NK-92	4-1BB.CD3ζ/2B4.CD3ζ	Lentivirus	[155]
T-cell leukemia	CD7	NK-92MI	CD28.4-1BB.CD3ζ	Electroporation	[156]
EBVA3C^+^T-cell lymphoblastic cells	EBNA3C	NK-92MI	4-1BB.CD3ζ	Retrovirus	[157]
Multiple myeloma	CD138	NK-92MI	CD3ζ	Lentivirus	[158]
Multiple myeloma	CD138; CD19	NK-92	CD28.4-1BB.CD3ζ	Lentivirus	[159]
Multiple myeloma	CS1	NK-92	CD28.CD3ζ	Lentivirus	[160]
Multiple myeloma	NKG2D	PB-NK	4-1BB.CD3ζ	Lentivirus	[161]
Acute myeloid leukemia	NKG2D	PB-NK	CD28.4-1BB.CD3ζ+IL-15	Electroporation	[162]
Acute myeloid leukemia	CD33	PB-NK	4-1BB.CD3ζ	Lentivirus	[163]
Acute myeloid leukemia	CD38	KHYG-1; PB-NK	CD28.CD3ζ	Electroporation	[164]
Acute myeloid leukemia	CD123	PB-NK	CD28.4-1BB.CD3ζ	Retrovirus	[165]
Acute myeloid leukemia	CD123	PB-NK	4-1BB.CD3ζ	Retrovirus	[166]
Acute myeloid leukemia	CD123	NK-92	4-1BB.CD28.CD3ζ	Retrovirus	[167]
Acute myeloid leukemia	NPM1c	PB-NK	4-1BB.CD3ζ	Lentivirus	[168]

### B-cell malignancies

CD19, ubiquitously expressed in the B lymphocyte lineage but with predominant expression on malignant B cells, is the most prevalent target exploited in CAR-NK based cellular therapy. NK-92 and primary NK cells have been engineered with a variety of anti-CD19 CARs, showing enhanced targeted killing ability toward an array of B-cell leukemia, B-cell lymphoma cells, and autologous leukemia blasts from patients [169–172]. According to these studies, the different intracellular domains of CAR can confer NK cells with varying degrees of cytotoxicity. First, the first generation anti-CD19 CAR incorporating CD3ζ endows NK cells with cytotoxic ability that is superior to that of DAP10-incorporating CAR counterparts [171, 172]. Second, the addition of one costimulatory molecule, such as 4-1BB or CD28, to the CAR can further augment the anti-leukemia response of primary NK cells [171]. However, it was found in NK-92 cells that CD19.4-1BB.CD3ζ CAR-NK-92 cells are less effective in tumor killing than NK-92 cells carrying CD19.CD3ζ CAR or CD19.CD28.CD3ζ [169]. Third, anti-CD19 CARs integrated with 2B4, an NK-associated activating receptor, can markedly overcome the tumor resistance of NK cells, showing robust killing activities toward autologous leukemia cells [170]. Identical outcomes were confirmed by anti-123.2B4ζ CAR-NK cells targeting AML cells and anti-CD5.2B4ζ CAR-NK cells targeting malignant T cells, and both CAR-NK cells showed more potent cytotoxicity toward target cells than their respective counterparts incorporating the 4-1BBζ component [155, 173]. In addition, CD19 CAR-NK-92 cells are sufficient to induce potent anti-lymphoma activity toward anti-CD20 antibody-resistant BNHL cells, offering a potential treatment alternative for some drug-resistant diseases [174]. Some clinical studies have demonstrated that the expansion and persistence of adoptive effector cells are correlated with clinical response [175, 176]. Researchers have pursued methods to increase CAR-NK cell longevity for better antitumor performance. Transgenic expression of secretory interleukin (sIL)-15, a critical cytokine supporting NK cell activation and expansion, on CAR-NK cells has been tested in the treatment of hematological tumors [26, 128, 143]. For example, the poor persistence of anti-CD19 CB-CAR-NK has been addressed by transducing the autocrine IL-15 gene into CAR, showing striking efficacy in eliminating patient-derived leukemia cells. To enhance the safety performance of autocrine IL-15 CAR-NK cells, an inducible caspase-9-based suicide gene (iC9) was also introduced to the CAR design, allowing pharmacological-mediated death of iC9/CAR.19/IL15 NK cells in case of adverse responses [128]. CD20 and FLT3 are also common targets in the immunotherapy of B-cell derived malignancies [144, 148, 149]. By virtue of the inherent ADCC ability mediated by FcγRIII of NK cells, Boissel et al. compared in vitro killing performance of NK cells mediated by two modes: a CD20-redirected CAR and anti-CD20 monoclonal antibodies (mAbs). The cytolytic response of CD20-CAR-NK cells significantly outperformed that of ADCC in the presence of a panel of NK-resistant CLL cells. CD20-CAR-NK cells significantly suppressed tumor growth and prolonged the survival of CLL-bearing mice [144].

Based on the inspiring outcomes from a large number of CD19 CAR-NK preclinical studies, CD19 is the most favorable target in clinical investigation for the treatment of B-cell derived lymphoma and leukemia. The aforementioned iC9/CAR.19/IL15 CB-NK cells were administered to the patients in incremental single doses. At a median follow-up of 13.8 months, the objective response rate was 73% [8, 11], and 7 out of the patients obtained a CR. The CAR-NK infusion did not induce any symptoms of adverse events. In addition, iC9/CAR.19/IL15 CB-NK cells were found to persist in patients for as long as 1 year by quantitative polymerase chain reaction, indicating their durable efficacy and potential [26]. As CB-NK cells and PB-NK cells are heterogeneous cells from which standardized products are difficult to generate, homogenous iPSC-derived CAR-NK cells are extensively utilized in clinical settings to treat a wide range of B-cell hematological cancers. Fate Therapeutics and Allife Medical Science and Technology are the two main forces producing CAR-NK products centered around iPSC-NK cells. FT596, a multiplexed iPSC-CAR-NK cell product from Fate, is engineered with a CD19-targeting CAR, a high-affinity CD16, and IL-15/IL-15Rα. FT596 was evaluated in a phase I clinical trial for patients with relapsed/refractory (R/R) B-NHL and CLL [177]. The disclosed data exhibited equivalent outcomes of FT596 with CD19 CAR-T cells in patients with CD19-positive B-cell malignancies, achieving a 71% ORR (10/14) and 50% CRR. In combination with rituximab (anti-CD20), FT596 had a stronger ability to kill CD19- and/or CD20-positive hematological tumor cells (NCT04245722). No evidence of CRS, ICANS, or GVHD was observed in these patients, showing the potential of iPSC-CAR-NK cells for widespread clinical use [178]. Soon, Fate Therapeutics will submit an Investigational New Drug (IND) application concerning the next-generation CD19-targeting iPSC-CAR-NK cell product with five innovative synthetic controls (FT522) in mid-2023 to further upgrade the strategies implemented on iPSC-NK cells.

### T-cell malignancies

The joint antigens between normal T cells and malignant T cells can induce the dysfunction and fratricide of post-infusion CAR-T cells [179]. NK cells, lacking multiple classic T-cell associated markers, can serve as an ideal alternative to treat aggressive T-lymphoid cancers [180]. CAR-NK cells have shown effectiveness in targeting several T-cell TAAs, including CD3, CD4, CD5 and CD7, among which CD5 is the investigational epicenter of targets for T-cell malignancy treatment. A third-generation CD5 CAR incorporating CD28 and 4-1BB costimulatory molecules was engineered in NK-92 cells and achieved potent anti-T-ALL responses with stable expansion ability [153]. Voynova et al. compared NK cells carrying an NK-cell-specific CAR framework (a CD8a hinge region, NKG2D transmembrane region, 2B4 costimulatory domain, and CD3ζ signaling domain) and T-cell-specific CAR framework (a hinge region, transmembrane and costimulatory domains of CD28, and a CD3ζ signaling domain) to target CD3- or CD5-positive T-ALL. The results showed that NK-specific CAR constructs can confer NK cells with more potent killing activities both in vitro and in a T-ALL xenograft mouse model. The author considered that CD5 is a better target than CD3 for CAR-based treatment of T-cell malignancies, as less antigen escape of T-ALL cells was found in the group treated with CD5 CAR-NK cells [152]. A phase I/II clinical study conducted by the M.D. Anderson Cancer Center is evaluating the safety and optimal dose of anti-CD5 CAR and IL15-transduced CB-NK cells in patients with relapsed/refractory T-cell malignances (NCT05110742). The results are currently not available. You and his colleagues engineered CD7-targeting CAR-NK-92MI cells by employing monovalent or bivalent CD7 nanobody VHH6 sequences in the CAR cassette. Both CD7-CAR-NK-92MI cells exhibited marked elevation of IFN-γ and Granzyme B in the exposure of T-ALL cell lines and primary tumor cells. In particular, bivalent CD7-CAR-NK-92MI cells had a superior inhibitory effect on T-ALL tumors in a mouse model [156]. In addition, CD7-CAR-engineered NK-92 cells have been clinically tested in patients with CD7-positive relapsed or refractory leukemia and lymphoma (NCT02742727).

### Myeloid malignancies

CAR-T-cell therapy has been extensively investigated for acute myeloid leukemia (AML) and multiple myeloma (MM), achieving impressive efficacy by targeting antigens such as BCMA, CD33, CD38, IL-3 receptor alpha chain (CD123), and CD138 [181]. Benefiting from sufficient research on CAR-T-cell therapy, recent paradigm-changing CAR-NK therapy has brought new hope in the treatment of myeloid malignancies.

CD123, the most-studied target in AML, has been targeted by several NK cell types engineered with CARs, including NK92, PB-derived, and CB-derived NK cells. Depending on the third- or fourth-generation anti-CD123 CARs, NK cell products can efficiently lyse AML cells in vitro and suppress tumors in AML xenograft mice, providing a firm foundation for further clinical transformation [165, 167, 182, 183]. CD38 is a typical molecule expressed on AML cells, NK cells, and some myeloid cells [184, 185]. The researchers used a natural CD38-low-expression NK cell line KHYG-1 to bear CD38-CAR, whose efficacy to lyse AML cells was greatly enhanced. To further minimize CAR-NK self-fratricide, the CD38 knockout technique was conducted on primary NK cells before anti-CD38 CAR transduction. Cytokine-induced memory-like (CIML) NK cells, characterized by enhanced and prolonged responses to tumor restimulation, have shown promising therapeutic effects on treating relapsed/refractory AML, according to recent clinical reports [104, 105, 186, 187]. Investigators have armed CIML NK cells with a CAR targeting a neoepitope (NPM1c), an AML-specific nucleophosmin-1 (NPM1) gene mutant, [146], resulting in more precise and robust killing as well as minimal on-target off-tumor effects [168]. The most commonly used virus-mediated CAR transfer modes require laborious virus production, and the virus quality is variable from lot to lot. An investigation applied a nonviral-form piggyBac transposon technology in the engineering of CAR NK cells to boost transduction efficiency and manufacturing stability. Utilizing this system, the team has successfully engineered PB-derived NK cells with an NKG2D-CAR cassette including the IL-15 gene, achieving synergistically enhanced anti-AML activity and improved in vivo persistence [162]. It is important to note that AML stem cells have also evolved mechanisms to evade NK cell recognition, such as the downregulation of NKG2D ligands, which may, in turn, limit the efficacy of CAR NK cell therapy [189].

CD138, a primary membrane protein on MM cells, is targeted by the first-generation CD138 CAR-NK-92MI cells. The results demonstrated enhanced and selective cytotoxicity toward CD138-positive MM cells in vitro and rapid regression of tumors in the subcutaneous injection mouse model [158]. CS1, which colocalizes with CD138 and ubiquitously exists on MM cells, is also a potential target for MM treatment. CS1 CAR-NK was analyzed in CS1-expressing MM cells and MM-bearing mice. Elevated secretion of cytokines and obvious tumor eradication were observed in a CS1-expression-dependent manner [160]. NKG2D, possessing a broad spectrum of ligand types on multiple cancer cells, was designed as a scFv component of CAR. The results showed that NKG2D CAR-NK cells could mediate strong antitumor responses in MM xenograft mice [161].

Clinical trials for T-cell-derived hematologic cancer are relatively limited. In 2018, a first-in-man phase I trial of CD33-CAR-NK-92 cells was initiated by Tang et al. to treat a small cohort of R/R AML patients (n = 3) (NCT02944162) [82]. Third-generation CARs, including CD28 and 4-1BB costimulatory domains, were transferred to NK-92 cells with a high transduction efficiency of up to 90%. Three patients received three incremental doses of CD33-CAR-NK-92 cells every other day, peaking at 5 × 10^9^ cells. Only moderate to high fevers or low-grade CRS were observed in the three patients, and these adverse effects were quickly relieved within two days. Then, sponsored by Xinqiao Hospital of Chongqing (China), a phase I clinical trial of CD33-CAR-NK-92 cell therapy was initiated in 2021 to evaluate the safety and efficacy of CAR-NK-92 cells in combination with the cancer medication cytoxan and the antineoplastic drug fludarabine in AML patients. The results are still pending. FT576, another pipeline product of Fate Therapeutics, aims to treat patients with relapsed/refractory multiple myeloma. Similar to FT596, FT576 encompasses a BCMA-targeting CAR, high-affinity CD16 and IL-15/IL-15Rα. In addition, CD38 is knocked out to mitigate the negative response induced by NK cell fratricide. According to its interim phase I clinical data, FT576 as a monotherapy or a combination therapy with daratumumab (anti-CD38 antibody) shows effective anti-myeloma activity [190].

### CAR-NK cell therapy for solid tumors

The application of cellular adoptive immunotherapy for hematological tumors has shown encouraging outcomes, but obstacles remain in the treatment of solid tumors. NK cells possess favorable attributes, such as the ability to infiltrate tumors and intrinsic recognition and killing of tumor cells, attracting investigators’ enthusiasm for exploiting CAR-NK cells in the treatment of solid tumors. A large number of CAR-NK preclinical studies have been conducted to test CAR-NK cell therapy efficacy for a series of solid tumors, mainly breast cancer (BC), ovarian cancer (OC), glioblastoma (GBM), and some types of gastrointestinal malignancies (Table 2). A few CAR-NK cell studies have moved into the clinical stage, and these studies are summarized in Table 3.

**Table 2:** Overview of preclinical studies based on CAR-NK cell therapy for solid tumors

Disease	Target	NK cell source	Intracellular domain	Transduction methods	References
Glioblastoma	EGFR; EGFRvIII	NK-92; NKL	CD28.CD3ζ	Lentivirus	[81, 218]
Glioblastoma	EGFR; EGFRvIII	PB-NK	CD28.CD3ζ	Retrovirus	[205]
Glioblastoma	Her2	NK-92	CD28.CD3ζ	Lentivirus	[192]
Glioblastoma	GD2; NKG2D	NK-92	CD28.CD3ζ; DAP10.CD3ζ	Electroporation	[219]
Glioblastoma	B7H3	CB-NK	CD28.CD3ζ	Retrovirus	[220]
Breast cancer	B7H6	NK-92	NKp30. CD3ζ	Retrovirus	[221]
Breast cancer	Epcam	NK-92	CD28.CD3ζ+IL-15	Lentivirus	[222]
Breast cancer	Her2	NK-92	CD28.CD3ζ	Electroporation	[223]
Breast cancer	Her2	PB-NK	CD28.CD3ζ	Lentivirus	[194]
Breast cancer	EGFR	NK-92; PB-NK	CD28.CD3ζ	Lentivirus	[224]
Ovarian cancer	GPC3	iPSC-NK	CD28.4-1BB.CD3ζ	Lentivirus	[225]
Ovarian cancer	MSLN	NK-92	2B4.CD3ζ	Lentivirus	[207]
Ovarian cancer	MSLN	iPSC-NK; NK-92	2B4.CD3ζ	Lentivirus	[96]
Ovarian cancer	CD133	NK-92	CD28.4-1BB.CD3ζ	Lentivirus	[209]
Ovarian cancer	CD24; MSLN	NK-92	CD28.4-1BB.CD3ζ	Lentivirus	[210]
Ovarian cancer	CD44	NK-92	CD28.4-1BB.CD3ζ	Lentivirus	[226]
Ovarian cancer	FRα	NK-92	CD28.4-1BB.CD3ζ	Lentivirus	[208]
Prostate cancer	PSMA	NK92MI	NKG2D	Lentivirus	[227]
Prostate cancer	PSMA	NK-92	2B4.CD3ζ	Lentivirus	[228]
Prostate cancer	PSMA	NK-92	CD28.CD3ζ	Lentivirus	[229]
Pancreatic cancer	MSLN	NK-92	4-1BB.CD3ζ	Unknown	[230]
Pancreatic cancer	PSCA	PB-NK	CD28.CD3ζ	Retrovirus	[231]
Pancreatic cancer	FRα	NK-92	CD27.CD3ζ	Lentivirus	[212]
Colorectal cancer	CEA	NK-92MI	CD3ζ	Retrovirus	[217]
Colorectal cancer	EPCAM; EGFRvIII; Fzd	NK-92	CD28.CD3ζ	Lentivirus	[232]
Colorectal cancer	EPCAM	NK-92	4-1BB.CD3ζ	Lentivirus	[233]
Colorectal cancer	NKG2D	NK-92	DAP12; CD3ζ	Electroporation	[139]
Gastric cancer	MSLN	NK-92	2B4.CD3ζ	Lentivirus	[77]
Small cell lung cancer	DLL3	NK-92	2B4.CD3ζ	Lentivirus	[234]
non-small cell lung cancer	CD70	PB-NK	CD28.4-1BB.CD3ζ	Retrovirus	[235]
Lung adenocarcinoma	c-Met	NK-92	2B4.DAP10.CD3ζ	Lentivirus	[137]
Neuroblastoma	B7-H3	NK-92	CD28.CD3ζ	Lentivirus	[236]
Melanoma	B7-H3	NK-92	CD28.CD3ζ	Lentivirus	[237]
Multiple solid tumors	HLA-G	PB-NK	DAP12+iC9	Lentivirus	[238]
PSCA^+^ cancer	PSCA	YTS; PB-NK	DAP12	Lentivirus	[135]
Neuroblastoma	GD2	NK-92	CD3ζ	Retrovirus	[239]
Ewing sarcoma	GD2	PB-NK	4-1BB.CD3ζ	Retrovirus	[240]
Hepatocellular Cancer Cells	GPC3	NK-92	DNAM1.2B4.CD3ζ	Lentivirus	[241]
Hepatocellular Cancer Cells	GPC3	NK-92	CD28.CD3ζ	Lentivirus	[215]

**Table 3:** Overview of clinical studies of CAR-NK

Clinical trial identifier	Target	Cancer type	Cell source	Phase	First posted	Current status	Sponsors	Country
NCT02742727	CD7	CD7^+^ R/R leukemia/lymphoma	NK-92	I, II	2016	Unknown	PersonGen BioTherapeutics (Suzhou) Co., Ltd	China
NCT03559764	BCMA	R/R multiple myeloma	iPSC-NK	I	2018	Unknown	Allife Medical Science and Technology Co., Ltd	China
NCT03940833	BCMA	Multiple myeloma	NK-92	I, II	2019	Unknown	Asclepius Technology Company Group (Suzhou) Co., Ltd	China
NCT05008536	BCMA	Refractory multiple myeloma	CB-NK	I	2021	Recruiting	Sichuan Kelun-Biotech Biopharmaceutical Co., Ltd	China
NCT05182073	BCMA	Multiple myeloma	iPSC-NK	I	2022	Recruiting	Fate Therapeutics	United States
NCT05652530	BCMA	Multiple myeloma	Unknown	I	2022	Recruiting	Shenzhen Pregene Biopharma Co., Ltd	China
NCT00995137	CD19	Acute myeloid leukemia	PB-NK	I	2009	Completed	St. Jude Children’s Research Hospital	China
NCT01974479	CD19	Acute lymphoblastic leukemia	PB-NK	I	2013	Suspended	National University Health System, Singapore	Singapore
NCT02892695	CD19	R/R CD19^+^ leukemia and lymphoma	NK-92	I, II	2016	Unknown	PersonGen BioTherapeutics (Suzhou) Co., Ltd	China
NCT03056339	CD19	B-cell lymphoid malignancies	CB-NK	I, II	2017	Recruiting	M.D. Anderson Cancer Center	United States
NCT03690310	CD19	Refractory B-cell lymphoma	iPSC-NK	I	2018	Unknown	Allife Medical Science and Technology Co., Ltd	China
NCT03579927	CD19	CD19^+^Mantle cell lymphoma; recurrent diffuse large B-Cell lymphoma…	CB-NK	I, II	2018	Withdrawn	M.D. Anderson Cancer Center	United States
NCT03824951	CD19	Refractory B-cell lymphoma	iPSC-NK	I	2019	Unknown	Allife Medical Science and Technology Co., Ltd	China
NCT04245722	CD19	R/R B-cell lymphoma or chronic lymphocytic leukemia	iPSC-NK	I	2020	Recruiting	Fate Therapeutics	United States
NCT04639739	CD19	Non-Hodgkin lymphoma	Unknown	I	2020	Not yet recruiting	Chongqing Precision Biotech Co., Ltd	China
NCT04796688	CD19	Acute lymphoblastic Leukemia Chronic lymphoblastic leukemia; B-cell lymphoma	Unknown	I	2021	Recruiting	Wuhan Union Hospital, China	China
NCT04796675	CD19	Acute lymphocytic leukemia;chronic lymphocytic leukemia; non-Hodgkin lymphoma	CB-NK	I	2021	Recruiting	Wuhan Union Hospital, China	China
NCT04887012	CD19	B-cell non-Hodgkin lymphoma	PB-NK	I	2021	Recruiting	Second Affiliated Hospital, School of Medicine, Zhejiang University	China
NCT05020678	CD19	non-Hodgkin lymphoma; B-cell acute lymphoblastic leukemia; Large B-cell lymphoma and 7 more B-cell cancer	PB-NK	I	2021	Recruiting	Nkarta Inc	United States
NCT05379647	CD19	B-cell lymphoma; B-cell acute lymphoblastic leukemia	unknown	I	2022	Recruiting	Zhejiang University	China
NCT05645601	CD19	Adult R/R B-cell hematologic malignancies	unknown	I	2022	Recruiting	Beijing JD Biotech Co. LTD	China
NCT05472558	CD19	B-cell non-Hodgkin lymphoma	CB-NK	I	2022	Recruiting	Second Affiliated Hospital, School of Medicine, Zhejiang University	China
NCT05410041	CD19	Acute lymphocytic leukemia; chronic lymphocytic leukemia; non-Hodgkin lymphoma	unknown	I	2022	Recruiting	Beijing Boren Hospital	China
NCT05336409	CD19	R/R CD19^+^ B-cell malignancies; indolent non-Hodgkin lymphoma; aggressive non-Hodgkin lymphoma	iPSC-NK	I	2022	Recruiting	Century Therapeutics, Inc	United States
NCT05570188	CD19	B-cell lymphoma B-cell leukemia	unknown	I, II	2022	withdrawn	Kunming Hope of Health Hospital	China
NCT05654038	CD19	B-cell lymphoblastic leukemia/lymphoma	unknown	I, II	2022	Recruiting	920th Hospital of Joint Logistics Support Force of People's Liberation Army of China	China
NCT05673447	CD19	diffuse large b cell lymphoma	unknown	I	2023	Not yet recruiting	Nanjing Enricnk Biotech Co., Ltd	China
NCT02892695	CD19	R/R CD19^+^ Leukemia and lymphoma	NK-92	I, II	2016	Unknown	PersonGen BioTherapeutics (Suzhou) Co., Ltd	China
NCT03824964	CD19/CD22	Refractory B-cell lymphoma	iPSC-NK	I	2019	Unknown	Allife Medical Science and Technology Co., Ltd;Peking University Cancer Hospital & Institute	China
NCT05667155	CD19/CD70	B-cell non-Hodgkin lymphoma	CB-NK	I	2022	Recruiting	Second Affiliated Hospital, School of Medicine, Zhejiang University	China
NCT04023071	CD20	Acute myelogenous leukemia; B-cell lymphoma	iPSC-NK	I	2019	Recruiting	Fate Therapeutics	United States
NCT03692767	CD22	Refractory B-cell lymphoma	iPSC-NK	I	2018	Unknown	Allife Medical Science and Technology Co., Ltd	China
NCT02944162	CD33	R/R acute myeloid leukemia	NK-92	I, II	2016	Unknown	PersonGen BioTherapeutics (Suzhou) Co., Ltd	China
NCT05008575	CD33	Acute myeloid leukemia	Unknown	I	2021	Recruiting	Sichuan Kelun-Biotech Biopharmaceutical Co., Ltd	China
NCT05215015	CD33 CLL1	Acute myeloid leukemia	Unknown	I	2022	Recruiting	Imbioray (Hangzhou) Biomedicine Co., Ltd	China
NCT05092451	CD70	B-Cell lymphoma; myelodysplastic syndromes (MDS); acute myeloid leukemia	CB-NK	I, II	2021	Recruiting	M.D. Anderson Cancer Center	United States
NCT05574608	CD123	Acute myeloid leukemia refractory; acute myeloid leukemia recurrent	Unknown	I	2022	Recruiting	Beijing JD Biotech Co. LTD	China
NCT04614636	CD38/SLAMF7	Acute myeloid leukemia; multiple myeloma	iPSC-NK	I	2020	Recruiting	Affiliated Hospital to Academy of Military Medical Sciences	China
NCT03415100	NKG2D	Solid tumors	PB-NK	I	2018	Unknown	Third Affiliated Hospital of Guangzhou Medical University	China
NCT04623944	NKG2D ligands	R/R acute myeloid leukemia; refractory myelodysplastic syndromes	PB-NK	I	2020	Recruiting	Nkarta Inc	United States
NCT05247957	NKG2D	Relapsed or refractory acute myeloid leukemia	CB-NK	I	2022	Terminated	Hangzhou Cheetah Cell Therapeutics	China
NCT05213195	NKG2D	Metastatic colorectal cancer	unknown	I	2022	Recruiting	Zhejiang University	China
NCT05528341	NKG2D	R/R solid tumors	NK-92	I	2022	Recruiting	Xinxiang medical university	China
NCT02839954	MUC1	Advanced solid tumors	unknown	I, II	2016	Unknown	PersonGen BioTherapeutics (Suzhou) Co., Ltd	China
NCT03383978	HER2	Glioblastoma	NK-92	I	2017	Recruiting	Johann Wolfgang Goethe University Hospital	Germany
NCT03692663	PSMA	Metastatic Castration-resistant prostate cancer	iPSC-NK	I	2018	Recruiting	Allife Medical Science and Technology Co., Ltd	China
NCT03692637	Mesothelin	Epithelial ovarian cancer	iPSC-NK	I	2018	Unknown	Allife Medical Science and Technology Co., Ltd	China
NCT03940820 NCT03931720 NCT03941457	ROBO1	Solid tumor	Unknown	I, II	2019	Unknown	Asclepius Technology Company Group (Suzhou) Co., Ltd	China
NCT04630769	CD276	Ovarian cancer; fallopian tube adenocarcinoma; primary peritoneal cavity cancer	iPSC-NK	I	2020	Completed	Masonic Cancer Center, University of Minnesota	United States
NCT04847466	PD-L1	Gastroesophageal junction cancers; advanced head and neck squamous cell carcinoma	NK-92	II	2021	Recruiting	National Cancer Institute (NCI)	United States
NCT05410717	Claudin6	Stage IV ovarian cancer; refractory testis cancer	PB-NK	I, II	2022	Recruiting	Second Affiliated Hospital of Guangzhou Medical University	China
NCT05194709	5T4	Advanced solid tumors	Unknown	I	2022	Recruiting	Imbioray (Hangzhou) Biomedicine Co., Ltd	China
NCT05507593	DLL3	Small-cell lung cancer	Unknown	I	2022	Recruiting	Tianjin Medical University Cancer Institute and Hospital	China

Human epidermal growth factor receptor 2 (HER2) is highly expressed in breast cancer [191], GBM [192], and renal cell carcinoma (RCC) [193]; thus, it serves as an attractive target for CAR-engineered NK cells. Portillo et al. used healthy donor and patient-derived NK cells to carry Her2.CD28.CD3ζ CAR. Under the condition of immunosuppressive factors such as TGF-β and PGE2, Her2 CAR-NK cells had robust cytotoxic effects on a series of HER2-positive breast cancer cells, and minimal to no toxic effects on healthy tissue cells were found [194]. Oxidative stress is also an immunosuppressive factor in the TME that can induce the dysfunction and death of NK cells [195, 196]. Peroxiredoxin-1 (PRDX1), a critical element of antioxidative defense, is absent in NK cells [197]. The investigators constructed NK-92 cells with stable PRDX1 expression and then engineered anti-PD-L1 CARs, which can support NK cell function in the presence of oxidative stress and induce potent killing of PD-L1-positive breast cancer cells [198]. In addition, NK cells that co-expressed anti-HER2-CAR and soluble PD-1 (designated sPD-1-CAR-NK cells) could suppress the interactions of PD-1/PD-L1 and significantly augment immunological anticancer efficacy in PD-L1^+^Her2^+^ breast cancer cells [199]. The first clinical trial of CAR-NK cells in Germany deployed Her2 CAR-NK-92 cells harboring CD28 and CD3ζ signaling domains (NK-92/5.28.z cells) to target recurrent HER2-positive glioblastoma [193, 200] (NCT03383978). The main objective was to evaluate the safety and tolerability of NK-92/5.28.z cells. NK-92/5.28.z cells were injected into the resection cavity during surgery in the dose-escalation scheme. To date, no toxicity has been observed at any dose.

EGFR, which is closely associated with tumor progression and migration, is expressed in 50% of GBM patients [201]. Intracranial injection of anti-EGFR.CD28. CD3ζ CAR-NK cells in the GBM mouse model showed obvious tumor repression and extended the overall survival rate. However, GBM tumors with EGFR amplification are often accompanied by a self-active EGFR mutant form, EGFRvIII, which contributes to heterogeneity and treatment resistance [202]. In 2018, Murakami et al. introduced an EGFRvIII-targeted CAR into the novel NK cell line KHYG-1, named EvCAR-KHYG-1 cells. Appreciable antitumor ability was observed in an EGFRvIII-dependent manner when cocultured with the GBM cell line U87MG [203]. To facilitate the homing of CAR-NK cells to the EGFRvIII-expressing tumor site, EGFRvIII-specific CARs with concomitant expression of the chemokine receptor CXCR4 on NK cells displayed redirected and evident migration to GBMs and significantly enhanced the mouse survival time in comparison with EGFR-CAR-NK alone [204]. In addition to CAR-NK cell monotherapy, combined therapies can also achieve outstanding effects. For example, taking advantage of the effective tumor-lysing ability of oncolytic viruses (OVs), an IL-15/IL-15Rα complex fusion protein was engineered on an OV (OV-IL15C) to synergistically favor the cytotoxicity, persistence, and infiltration of EGFR-CAR-NK cells. Better tumor regression-inducing capability was achieved than with either monotherapy [205].

CAR-NK cells are also employed in targeting HER2 [206], mesothelin (MSLN) [207], folate receptor-α (FRα) [208], and CD133 [209] to treat ovarian cancer. Mesothelin (MSLN)-targeted CAR-NK92 cells were identified as having potent cytotoxicity against MSLN-positive ovarian cell lines such as OVCAR-3 and SKOV3. The conspicuous tumor elimination ability in both subcutaneous and intraperitoneal tumor models further indicated that MSLN is a reliable target for future ovarian cancer treatments. Moreover, a novel dual-CAR was engineered into NK92 cells for the simultaneous targeting of MSLN and CD24; these cells could promote the apoptosis of both ovarian cell lines and primary ovarian tumor cells, and off-target effects were greatly minimized [210]. In clinical settings, anti-MSLN CAR-NK cells were developed by Allife Medical Science and Technology Company for patients with epithelial ovarian cancer (NCT03692637). This study is not yet recruiting, and no additional information is available. CAR-NK therapy targeting claudin-6 for ovarian cancer treatment is in the phase I/II stage of clinical trials, and the results are still pending [211].

Gastrointestinal cancers such as pancreatic cancer (PC), hepatocellular carcinoma (HCC) and colorectal cancer (CRC) have also been largely evaluated in recent years. Pancreatic ductal adenocarcinoma (PDAC) accounts for 90% of all kinds of PC, and its immunosuppressive stroma greatly hinders the infiltration of immune cells. Through bioinformatic integration of patient-derived samples, folate receptor α (FRα) and death receptor 4 (DR4) have been identified as optimal targets in the treatment of PDAC. A study confirmed that FRα-redirected CD27.CD3ζ CAR-NK92 cells with surface-displayed TRAIL vastly enhanced tumor-selective apoptosis both in vitro and in xenograft mice [212].

GPC3 is overexpressed in hepatocellular carcinoma (HCC) cells but is undetectable in normal tissues [213, 214]; therefore, it represents an ideal immunotherapeutic target. Hu9F2, a humanized anti-GPC3 scFv, was incorporated into CAR to redirect NK92 cells, killing HCC cell lines with gradually decreasing GPC3 expression. Selective lysis of GPC3-positive HCC cells was observed in vitro and in multiple HCC xenograft mouse models [215]. CD147 is expressed in several cell types and is particularly upregulated in pathological cells, including HCC cells. To minimize the on-target/off-tumor toxicity of CAR-NK cells, Tseng et al. used a logic-gated (log) synthetic notch to regulate the killing of dual-targeting (GPC3 and CD147) CAR-NK cells. Specifically, synthetic notch-mediated CAR-NK cells could recognize and eliminate double-positive (GPC3^+^CD147^+^) HCC cells but remained inactivated against single-positive (GPC3^−^CD147^+^ or GPC3^+^CD147^−^) HCC cells. In a human CD147-transgenic mouse model, the median survival time of mice treated with dual-targeted CAR NK cells was significantly extended, and there was no additional on-target/off-tumor effect during the observation period [216].

NKG2D CAR-NK cells incorporating the cytoplasmic domain of DAP12 (NKG2Dp CAR) have been employed to treat colorectal cancer (CRC), resulting in effective elimination of CRC cell lines and prolonging the survival time of CRC-bearing mice model [139]. Furthermore, the researchers successfully tested the feasibility of NKG2D CAR-NK cells in three patients with chemotherapy-refractory metastatic colorectal cancer (NCT03415100). Multiple doses of NKG2Dp CAR-NK cells were intraperitoneally infused into two patients with severe CRC burdens; the NKG2Dp CAR-NK cells contributed to a significant reduction in the volume of ascites and tumor regression. In the third patient with metastatic colon cancer in the liver, intraperitoneal infusion together with ultrasound-guided percutaneous injections of NKG2Dp CAR-NK cells induced obvious tumor regression in the liver. Additionally, another clinical trial of NKG2D CAR-NK cell therapy was initiated in 2022 to assess its safety and efficacy in patients with refractory metastatic colorectal cancer (NCT05213195). The detailed data have not been disclosed. Carcinoembryonic antigen (CEA) is another CRC target. CAR-NK-92MI cells have potent cytotoxicity against CEA-positive CRC cells. The administration of the histone deacetylase inhibitor sodium butyrate (NaB) or the methylation inhibitor 5-azacytidine (5-AZA) can induce CEA expression on CRC cells, which further enhances anti-CEA CAR-NK-92MI cell-induced cytotoxicity. This combination therapy showed potential to be clinically applied in terminal-stage colorectal cancer treatment [217].

The remaining CAR-NK cells in clinical trials target a diverse set of antigens, such as PSMA for prostate cancer, DLL3 for small-cell lung cancer, and roundabout homolog 1 (ROBO1) and MUC1 for advanced solid cancers. In summary, the number of clinical studies on solid tumors is still limited. The results of these clinical studies are still awaited to determine the persistence and durable response of CAR-NK cells. Additionally, it is noteworthy to observe whether the risk of tumor escape in CAR-NK cell therapy would be lower than that in CAR-T-cell therapy, as CAR-NK cells possess CAR-independent innate killing ability.

## Strategies to address the limitations of CAR-NK cell therapy

**Fig. 4:**
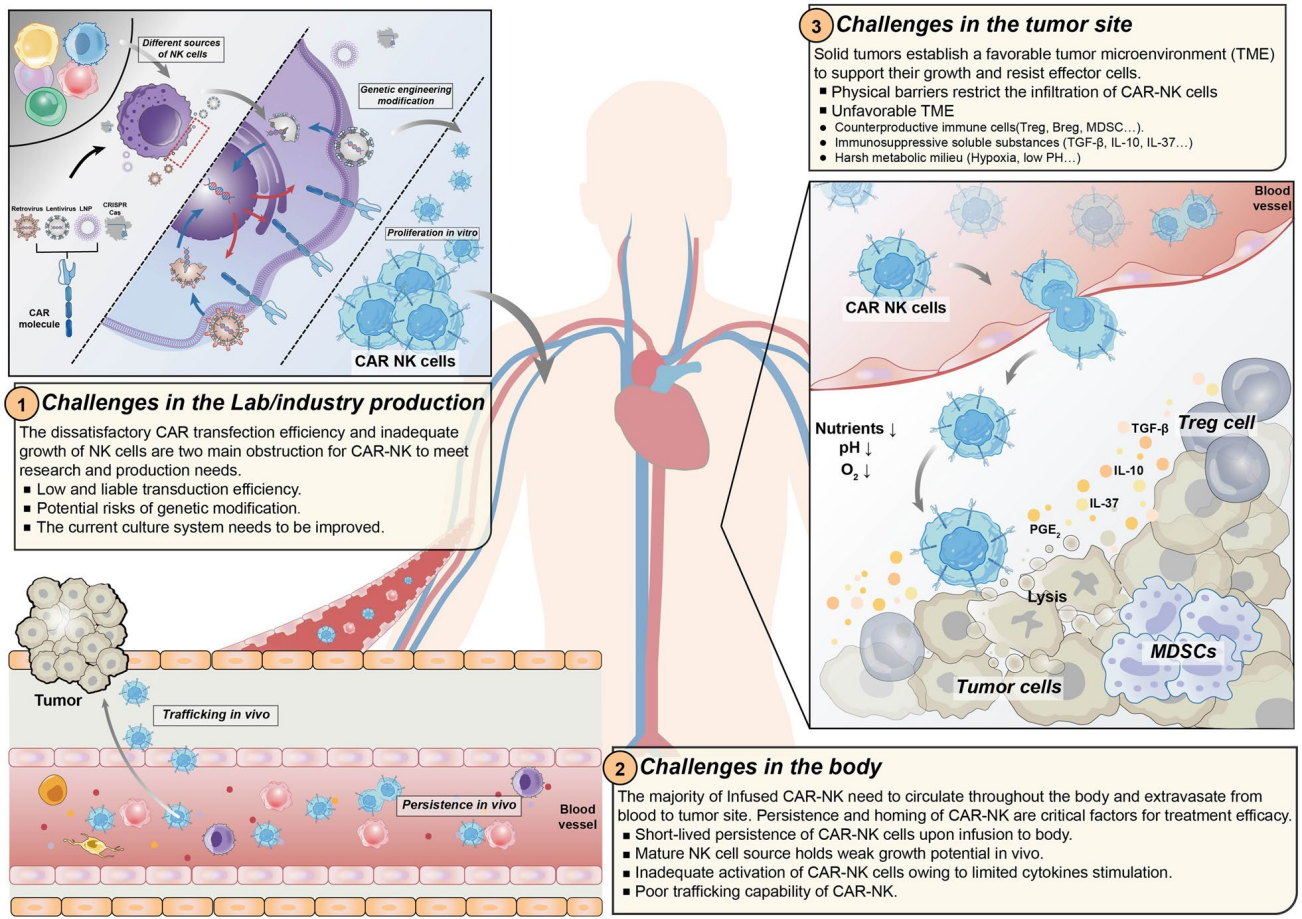
The challenges of CAR-NK existing in the process from lab production to tumor infiltration. The unsatisfactory CAR transduction efficiency and limited proliferation ability add barriers to CAR-NK production. Multiple approaches including virus-mediated and non-viral-mediated transduction have been utilized to boost CAR expression and stability. The ex-vivo expansion are mainly stimulated by cytokines or feeder cell system with limited potential. Upon infusion into the body, the trafficking and infiltration abilities are impeded by the disruptive chemokines/chemokine receptors axis in the dysregulated tumor vasculature. In tumor bed, suppressive cells (Treg cells, Breg cells and MDSCs) and soluble inhibitory cytokines (TGF-β, IL-10 and IL-6) can disrupt NK cell effector functions. The harsh TME owing to the nutrient deficiency, hypoxia, and acidic conditions can further suppress and dampen NK activities

Despite the remarkable outcomes of CAR-NK cells in treating a series of tumors, there are still obstacles in the field. Here, we focus on the major challenges that significantly restrict the production and therapeutic efficacy of CAR-NK cells (Fig. 4), sketching a developmental path for broader application.

### CAR-NK cell transduction efficiency

The most commonly used transduction systems in CAR-NK cell therapy are also lentiviruses (LVs) and retroviruses (RVs) [242]. However, LV and RV-mediated CAR transduction into NK cells has not shown the same satisfactory efficiency as that into T cells [140]. Other viruses being investigated for transduction, such as vaccinia and adenoviruses, are not suitable for NK cells, as they can alter the cytotoxic phenotype of NK cells and possess weaker transduction capability [243].

To enhance viral-mediated transduction efficiency, some small molecular compounds are used to reduce the repulsion of NK cells to foreign viral particles [244]. Negative charges on the surface of both the virus and the target cell are detrimental for transduction [245]. Thus, some cationic polymers, such as protamine sulfate, polybrene, and dextran, can promote transduction efficiency by positively charging cells [244, 246]. RetroNectin, a recombinant human fibronectin fragment, can vastly enhance the transduction efficiency of NK cells by colocalizing viruses and cells in close proximity [141, 247]. Similarly, Vectofusin-1 can promote CAR transfer by augmenting the adhesion of the virus to the cellular plasma membrane. There is no consensus on which is a superior transduction enhancer [248]. BX795 is an inhibitor of TBK1/IKKɛ, which are critical kinases involved in antiviral response signaling pathways [249, 250]. This functional inhibitor has been reported to greatly enhance gene transfer efficiency in primary immune cells [251, 252]. Other compounds have been utilized in various immune cells to improve transduction, such as phytohemagglutinin (PHA) [253], prostaglandin E2 (PGE2), and phorbol 12-myristate 13-acetate (PMA), but further mechanistic description is lacking.

Vesicular stomatitis virus (VSV) G-protein has been widely used for pseudotyping lentiviruses, as VSV-G has broad cell tropism [254]. Low-density lipid receptor (LDL-R), the main receptor of VSV-G, has a low level of expression on NK cells [255, 256]. This may be an explanation for the low transduction efficiency of VSV-G pseudotyped lentiviruses (VSV-G-LVs) into NK cells. Statins are widely prescribed medications for CLL patients, but they were recently identified with the function of upregulating LDL-R expression on immune cells, including NK cell lines and primary NK cells [256, 257]. Theoretically, transduction efficiency can be enhanced through statin administration. However, not all statins are suitable for boosting VSV-G-LV transduction efficiency, as most statins can negatively affect cell viability. In a comparative study, rosuvastatin was found to be the most potent substance to augment transduction, and its suppressive effect can be reversed in the presence of GGPP [256]. In addition to regulating LDL-R expression on target cells, employing other glycoproteins to pseudotype the viral vectors is also a feasible strategy to improve virus-mediated transduction efficiency [118]. The virtually unanimous conclusion is that the expression level of lentivirus receptors on target cells has a positive correlation with the transduction efficiency of the virus. BaEV, MV-, and RD114-pseudotyped viruses have been tested in NK cell transduction, and BaEV showed the best performance in large part due to the high expression of its receptors on NK cells [171, 255, 258].

Due to the risk of viral insertional mutagenesis and quality variability in large-scale viral production [259, 260], nonviral transduction methods have gained more attention in recent years.

Electroporation is the most commonly used nonviral transfection method. It is considered a safer transduction approach because it induces short-term gene expression [261, 262]. Electroporating DNA into cells showed a limited transduction rate, but superior performance was presented in electroporating mRNA, which can reach up to a 95% transduction rate with minimal damage to cell viability [149, 263, 264]. The transfection efficiency is significantly enhanced in electroporating NK-92 cells but with limited improvement in transducing CB-NK and PB-NK cells [263, 265–267]. Several preclinical results have demonstrated the efficacy of electroporation-based CAR-NK cells in the treatment of both solid and hematologic tumors [76, 139, 268]. Nucleofection is a transduction method based on electroporation, depending on the specific electric pulse used to directly deliver DNA into the cell nucleus, regardless of the cell division phase [269]. Nucleofection has been employed to induce CAR-NK cells to express a range of CARs targeting ROR in solid tumors [270] or CD20 in hematologic tumors [148, 149]. However, the transience of CAR expression induced by electroporation-based methods necessitates the transfusion of cell products into patients within seven days. The cell membrane damage and cell death caused by electric pulses are also major concerns for the continuous expansion of electroporation in clinical settings [271].

Two nonviral transposon systems, namely, Sleeping Beauty (SB) and PiggyBac (PB), can provide long-term transgene expression by inserting foreign genes by “a cut-and-paste” mechanism [272–275]. SB and PB systems have several advantages over virus-mediated transduction approaches: (1) more random gene integration; (2) a large capacity for foreign genes; and (3) cost-effective production of the basic components [276, 277]. These attributes make the SB and PB systems attractive tools for CAR-based therapy. In recent years, transposon systems have been mainly applied to generate CAR-T cells in preclinical and clinical settings [278–282] but are in the minority of systems used in the CAR-NK engineering field. NK-92 cell lines were easier to engineer with CARs by transposon-based methods, and the resulting products showed effective antitumor responses [138, 283]. Recently, investigators have successfully engineered PB-NK cells with NKG2D CAR and the IL-15 gene using the PB system [162]. There are also some drawbacks to transposon-mediated methods, such as uncontrolled transposition events and transgene remobilization in target cells [284, 285]. Additionally, the transfer of transposon components (such as transposage and gene vectors) needs to be promoted by a virus or electroporation, which can lead to the negative effects mentioned above [272, 286].

CRISPR/Cas9 is a potent genetic modification technique that has been widely applied in cellular immunotherapy [287]. The CRISPR/Cas9 system generally consists of two components: a single guide RNA (sgRNA) and Cas nuclease protein [288]. The precise and highly efficient gene-editing process is initiated through the recognition of specific gene loci by the sgRNA, followed by interaction with Cas9. CRISPR/Cas9 has been utilized in the CAR-T therapy field to address multifaceted issues such as generating allogeneic CAR-T cells and overcoming CAR-T cell exhaustion and the negative factors of the TME [289–291]. Similarly, CRISPR/Cas9 was initially adopted to disrupt or insert functionally relevant genes to improve CAR-NK cell performance. Researchers have successfully knocked out CD38 to prevent NK cell fratricide [164] and conducted triple editing (disruption of ADAM17 and PDCD1, knock-in of CD16), achieving high manipulation efficiency and enhanced function [292]. More recently, CRISPR/Cas9 has also been utilized to realize highly efficient and locus-specific CAR transduction into immune cells. Directing CD19 CAR to the T-cell receptor α constant (TRAC) locus by CRISPR/Cas9 resulted in consistent CAR expression in human peripheral blood T cells as well as improved effector responses [293]. One group combined CRISPR/Cas9 with an adeno-associated virus (AVV)-mediated gene-delivery approach to insert anti-CD33 CAR to a safe-harbor locus of primary NK cells, acquiring a mean expression of 68% CAR-positive NK cells and enhanced anti-AML activity [294].

Other emerging transduction strategies also include lipid nanoparticle (LNP)- and charge-altering releasable transporter (CART)-based transduction. LNPs and CARTs serve as protective carriers of nucleic acids, infusing into the cells without degradation by the nucleases. Once entering the cell cytosol, these substances can transform into a positively charged state to allow the release of internal mRNA and then proceed with protein expression. These strategies have been demonstrated to be effective in anti-CD19 CAR transduction into NK cells [295–297]. Altogether, these strategies have vast potential as genetic engineering tools in cellular immunotherapy but are still in their infancy of development. More investigations are required to test the safety performance and persistence of these cell products.

### CAR-NK cell expansion and persistence

Large amounts of NK cells are required for clinical therapy to achieve sufficient responses. However, the weak in vitro expansion of NK cells significantly hinders CAR-NK cell production and broad application. Autologous NK cells from patients account for a smaller proportion of cells in PB, causing additional difficulty for NK cell expansion [298]. A common expansion method relies on a series of cytokines for stimulation, such as IL-2, IL-12, IL-15, IL-18, and IL-21 [299]. A specific cytokine cocktail can tune NK cells to a particular phenotype. For example, the combination of IL-12, IL-15, and IL-18 can facilitate the generation of memory-like NK cells, which exhibit optimal in vivo persistence and antitumor activity [103, 104, 187, 300]. Nevertheless, the proliferation of cytokine-induced NK cells is associated with limited fold changes. Furthermore, solely depending on cytokines, NK cells easily become cytokine-susceptible and cytokine-addicted, which may raise a major concern for in vivo persistence and vitality in the absence of abundant cytokines [301].

Feeder cells serve as large-scale culture systems that combine cytokine stimulation and receptor-mediated activation [299]. K562 cells are representative feeder cells. Other cells, such as the Epstein‒Barr virus-transformed lymphoblastoid cell line (EBV‑LCL), 721.221, and PBMCs, are also exploited as feeder cells [205, 302, 303]. They are generally engineered to express membrane-bound cytokines (IL-2, IL-15, and IL-21) and/or ligands of NK activating receptors (4-1BBL, OX40L, and HLA-E), which can synergistically promote persistent expansion and antitumor activity [303–307]. K562 cells expressing IL-21 and 4-1BBL have been tested clinically and are considered safe in patients [303]. Compared to the sole cytokine culture system, feeder cells can significantly extend the number of fold changes and alleviate the dysfunction and apoptosis of NK cells induced by cytokine deficiency post-infusion. However, the majority of feeder cells are derived from cancer cell lines and thus must be lethally irradiated prior to infusion. Many concerns are arising about whether surviving feeder cells and other unknown factors could pose potential risks in the context of a complex body environment.

To circumvent the administration of feeder cells to support activation and proliferation, several groups are endeavoring to manipulate CAR plasmids incorporating cytokine transgenes to facilitate expansion and persistence [248–250]. The expression form of cytokine gene cassettes can be either membrane-bound or constitutively autocrine. A team from MD Anderson Cancer Center managed to engineer CAR-CB-NK cells to express IL-15 in a constitutively autocrine manner, demonstrating enhanced proliferation and in vivo persistence. There were no signs indicating elevation of systematic IL-15 or other toxicities [26, 128]. In another study, ectopic expression of IL-15 significantly prolonged the persistence of NKG2D CAR- NK cells both in vitro and in vivo. Additionally, the effector function of CAR-PB-NK cells was also significantly facilitated in AML mouse models [162].

### The trafficking and infiltration capabilities of CAR-NK cells

It is easier for infused CAR-NK cells to come into contact with hematological cancer cells in circulating peripheral blood; however, in their trafficking to solid tumor sites, multifaceted obstacles are encountered. The trafficking ability and infiltration amounts of NK cells have prognostic value for improved clinical outcomes [309–311].

To surmount the anatomical barriers in the treatment of solid tumors, orthotopic injections such as intraperitoneal injections, anterior prostatic lobe injections, and other ultrasound-guided injections have demonstrated effective tumor elimination in CAR-NK cell therapy without tissue damage [139, 192, 312]. In a phase I clinical trial in 9 patients with recurrent HER2-positive GB, NK-92/5.28.z cells targeting Her2 were injected into the wall of the resection cavity during relapse surgery. The disease progression of 5 patients was suppressed, lasting for 7 to 37 weeks, and no signs of dose-limiting toxicities were observed, demonstrating the feasibility and safety of intracranial injection of HER2-targeted CAR-NK cells [313].

The commonly used injection method is intravenous (i.v.) injection. CAR-NK cells need to extravasate from the blood and migrate to the solid tumor bed. This homing process is regulated by the dynamic chemokine receptor-and-ligand interactions between NK cells and tumor cells [314, 315]. Thus, increasing the expression of chemokine receptors on NK cells is a major strategy initially applied in NK cell-based therapies. A growing body of studies has equipped NK cells with chemokine receptors (CCRs), such as chemokine receptor chemokine (C-X-C motif) receptor 2 (CXCR2) [316, 317], CXCR4 [318] and CXCR7 [319], to match their cognate ligands expressed on tumor cells, and improved chemoattraction in the antitumor response of NK cells was shown. Concomitant expression of the CXCR1 or CXCR4 transgene on CAR-NK cells has also demonstrated enhanced migration to CD19^+^ hematological tumors [320], glioblastoma [321] and ovarian tumors [322]. The release level of chemokines in the TME varies greatly following different kinetics of the tumors, indicating the need for artificial infusion of sufficient chemokines to attract NK cells [318]. The feasible approaches include direct local administration of stimulation factors [318] or delivery of fusion proteins loaded with chemokine ligands [323]. The latter can release chemokine ligands upon engagement with tumor cells, thus facilitating the connection of chemokine receptor/ligand axes. However, these chemokine interactions are versatile and vary in different milieus, possibly causing a totally reversed effect (either promoting or diminishing) on the trafficking ability of NK cells [324, 325]. Thus, more efforts should be made to investigate the comprehensive mechanisms and responses of the chemokine interplay in the intricate context of the human body.

As hinted above, the complex TME is another major barrier that hinders the homing and function of CAR-NK cells. The obstructive factors existing in the TME network can be generally divided into three parts: counterproductive cells, immunosuppressive soluble substances, and a harsh metabolic milieu [180]. In the normal environment of tissues, immune cells such as myeloid cells, regulatory T (Treg) cells, or regulatory B (Breg) cells can provide positive feedback to the proinflammatory cytokines secreted from NK cells. They similarly produce cytokines such as IL-12, IL-15, and IL-18 to promote NK cell growth, maturation, and functionality [53, 326]. However, the formulation and exacerbation of the TME in solid tumors render some immune cells, including Treg cells, myeloid-derived suppressor cells (MDSCs), and tumor-associated macrophages (TAMs), to have suppressive roles. Under TME pressure, NK cells can also be forced to transform to have suppressive phenotypes, which discount their activity and infiltration ability [325]. Traitorous immune cells release inhibitory cytokines such as transforming growth factor beta 1 (TGF-β), prostaglandin E2 (PGE2), and IL-10 or “absorb” cytokines that are favorable for NK cells (IL-2), both of which directly or indirectly impede NK cell response and survival [327–330]. Thus, circumvention of these negative modulators is critical for CAR-NK cell therapy efficacy in vivo [331]. Pharmacological interventions such as chemotherapies have been adopted to eliminate MDSCs and Treg cells [233, 332, 333]. Additionally, NK cells were manipulated with the NKG2D.ζ CAR construct to target NKG2DL-expressing MDSCs, and cytotoxicity toward MDSCs in the xenograft TME model was observed. NKG2D.ζ CAR-NK cells have been tested in clinical studies to show that they can effectively eliminate intertumoral MDSCs in neuroblastoma patients and facilitate the infiltration and efficacy of infused CAR-T cells [334].

Excessive TGF-β is produced by immunosuppressive cells and tumor cells themselves in the TME. Pathologic levels of TGF-β are often correlated with serious disease progression and an impaired immune system [335–338]. Multiple strategies have been employed to disrupt TGF-β signaling to preserve NK cell therapy efficacy. Genetically engineering negative TGF-β receptors into NK cells [339–341] or knocking out TGF-β signaling-related downstream mediators on NK cells [342] successfully diminished or eliminated the blocking effects of TGF-β on NK cells. In vivo studies have identified enhanced NK cytolytic efficacy in GBM-engrafted mice after silencing TGF-β signaling. Additionally, molecular kinase inhibitors [341, 343, 344] and monoclonal antibodies [345] targeting TGF-β have exhibited equivalent antagonistic effects and improved the performance of infiltrating NK cells. Other TME-abundant suppressive soluble factors, including adenosine, PGE2, IL-10, and IL-37, are likewise potential targets to reverse the unfavorable TME [346, 347]. Adenosine is a metabolic byproduct in response to hypoxia in the TME, and its accumulation can paralyze NK cell functional activity [348]. The mainstream strategies to circumvent the negative effects of adenosine are targeting the hypoxic ectoenzyme CD73 [219, 349–352] and/or knocking out A2A receptors expressed on immune cells [352–355]. The results showed a plunging concentration of adenosine in the TME as well as the revival of NK cells. A triple-functional NK was manipulated with a locally released CD73 antibody fragment concomitant with dual-targeting (NKG2D and GD2) CAR expression to target GBM [219]. Enhanced cytolytic ability and persistence of intratumoral NK cells have been observed. Regional regulation of adenosine did not cause metabolic disorders in the whole body. Lever aging eminent preclinical performances, successional clinical trials using CD73 blocking antibodies have been carried out to overcome the dilemma of treating solid tumors (NCT04148937, NCT03454451, and NCT03616886).

Hypoxia, a typical hallmark of the TME, develops as a result of the malignant outgrowth of barely vascularized solid tumor tissues [356]. Restricted oxygen concentrations and deficient nutrients in solid tumor regions induce the downregulation of NK cell functional molecule expression (such as activation/inhibitory receptors, cytokines and death receptors) and substantially suppress their killing performance and migration ability [357, 358]. Nevertheless, relying on the hypoxic TME, tumor cells can escape immune cell monitoring and attack [359, 360]. Thus, mitigating intratumor hypoxia is a potential strategy to improve the treatment of solid tumors. Multiple pharmacological and physical strategies have been widely investigated in a series of preclinical and clinical studies to directly adjust the hypoxic state [361–367]. For example, hypoxia-activated prodrugs (HAPs) [368] or inhibitors targeting the hypoxia-inducible factor (HIF) protein family may be applied, or patients may even be physically exposed to a hyper oxygenated environment [369]. These strategies may provide directions to ameliorate and transform infiltrating-tumoral CAR-NK cell functional and metabolic exhaustion. We can also draw inspiration from a recent novel hypoxia-sensing CAR-T cell structure, which made full use of deleterious hypoxia as a switch to favor itself, launching a CAR-mediated killing procedure [370, 371]. This approach may establish a good pattern for the future design of CAR-NK cells to overcome hypoxia. Another unfavorable condition is the low pH in the TME. As a result of hypoxia, more anaerobic glycolysis reacts to support the activity of tumor cells; thus, increasingly accumulated lactic acid can damage the functionality of NK cells and promote suppressive immune cells [372]. The depletion of excessive metabolites is a direct strategy. However, it is noteworthy that preventing excessive immune metabolite modulation and maintaining a physiologic balance of the inner environment would be critically important for NK cell function.

## Conclusion and future perspectives

Paradigm shifting CAR-T-cell therapy has pioneered the development of the CAR technique and yielded promising outcomes in treating hematological tumors. Benefiting from the technologies and valuable lessons learned from CAR-T cell therapy, CAR-NK cell therapy has advanced rapidly with continuous innovations. Preclinical and early clinical outcomes have demonstrated the vast potential of CAR-NK cells as “off-the-shelf” products for cancer treatment. To date, numerous strategies have been applied in CAR-NK cell therapies to address the challenges discussed above, and satisfactory outcomes have been observed in preclinical studies. However, some of these strategies are difficult to translate into clinically approved procedures, such as the systematic infusion of NK-cell-stimulating cytokines. In contrast, multiplexed CAR-NK cell design systems or combinatorial approaches based on radiotherapy and other FDA-approved drugs may hold great potential to overcome the barriers in the CAR-NK cell therapy field and provide clinical benefit. With the development of cutting-edge technologies such as single-cell RNA sequence analysis (scRNA-seq), we have access to elucidating key parameters associated with CAR-NK cell biological function and therapeutic efficacy, which may provide investigators and clinicians with critical insights into how to optimize the promise of NK cell-based cancer therapy. In the coming years, clinical translation-oriented research and in-depth clinical testing of CAR-NK cell therapies are urgently needed to determine their potential market authorization.

## Data Availability

Not applicable.

## Abbreviations

ACT: Adoptive cell therapy
ADCC: Antibody-dependent cellular cytotoxicity
ALL: Acute lymphoblastic leukemia
AML: Acute myeloid leukemia
BCMA: B cell maturation antigen
CAR: Chimeric antigen receptor
Cas9: CRISPR-associated system 9
CCR: Chemokine receptors
CEA: Carcinoembryonic antigen
CLL: Chronic lymphocytic leukemia
CIML: Cytokine-induced memory-like NK cells
c-Met: Cellular-mesenchymal epithelial transition factor
CR: Complete remission
CRS: Cytokine release syndrome
CRISPR: Clustered regularly interspaced short palindromic repeats
CSC: Cancer stem cell
CSPG4: Chondroitin sulfate proteoglycan 4
DLBCL: Diffuse large B-cell lymphoma
DLL3: Delta-like ligand 3
EBV-LCL: Epstein–Barr virus-transformed lymphoblastoid cell line
EGFR: Epidermal growth factor receptor
EGFRvIII: EGFR variant III
EPCAM: Epithelial cell adhesion molecule
EPHA2: EPH Receptor A2
ER: Estrogen receptor
FasL: Fas ligand
FcγRIII: Fragment crystallizable receptor III
FDA: US Food and Drug Administration
FLT3: FMS-like tyrosine kinase 3
FRα: Folate receptor alpha
Fzd: Frizzled
GM-CSF: Granulocyte-macrophage colony-stimulating factor
GVHD: Graft-versus-host disease
GPC3: Glypican-3
Her2: Human epidermal growth factor receptor 2
hESC: Human embryonic stem cells
HLA: Human leukocyte antigen
IFN-γ: Interferon-γ
iPSC: Induced pluripotent stem cell
IS: Immunologic synapse
iKIRs: Inhibitory killer cell immunoglobulin-like receptors
mAb: Monoclonal antibody
M-CSF: Macrophage colony-stimulating factor
MHC: Major histocompatibility complex
MSLN: Mesothelin
MM: Multiple myeloma
NCR: Natural cytotoxicity receptor
NK: Natural killer
NSG: NOD scid gamma
PB: Peripheral blood
ORR: Objective response rate
PR: Progesterone receptor
PSMA: Prostate-specific membrane antigen
PSCA: Prostate stem cell antigen
PSMA: Prostate specific membrane antigen
RCC: Renal cell carcinoma
SCLC: Small cell lung cancer
scFv: Single-chain antibody variable fragment
scRNA-seq: Single-cell RNA sequence analysis
SLO: Secondary lymphoid organs
TAA: Tumor-associated antigen
TF: Tissue factor
TGF-β: Transforming growth factor-beta
TME: Tumor microenvironment
TNBC: Triple‑negative breast cancer
TRAIL: TNF-related apoptosis-inducing ligand
UCB: Umbilical cord blood
